# Pivot Teeth

**Published:** 1873-01

**Authors:** F. L. Hunter


### ARTICLE IV.

### *Pivot Teeth.*

BY F. L. HUNTER, D. D. S.

Many and ingenious methods have been resorted to, to make efficient what seems, under some circumstances, so desirable, viz: Pivot Teeth. In but few instances, however, and then only under the most favorable circumstances, with the nicest manipulation, have they been successful, since the point of contact of root and crown is never a union close enough to prevent its being a mere starting point for softening or decay. As a remedy for that evil I beg leave to offer the following to my professional brethren with the hope of its usefulness.

Instead of adapting a crown with a fixed pin, of either wood or metal, to a root, insert a metal pin in the root; make a filling of gold on the end of the root, and adapt your crown to that. You will only expose what appears a small filling along the margin of the gum. Our own method is to insert an eighteen karat pin (platinum alloy) firmly into the root by means of threads cut on that part of the wire to be inserted, having the other end cleft with a watch spring saw, and protruding sufficiently to occupy the hole in the crown; we now adjust the crown, which, however, should have been measurably investigated before the wire was inserted, by means of a stick loosely inserted in crown and root. The wire having been finally screwed into the root, and a proper crown selected and adjusted, (of course the end of the root should have been filed and polished, but the filling should not be carried into the gum, unless compelled to do so in removing decay,) remove the crown, and enter two or three of Dr. Mack's short retaining screws. Now cut a number of pieces from the heaviest gold—say No. 480—a little longer than the end of the root, with holes punched to correspond with or pass over the pins in the root; lay on one after another, condensing with a smooth-faced instrument, occasionally paying particular attention to condensing small pieces around the pins. The concavity usually given the root (though it should not be so great) should be maintained on the surface of the filling; which, having been raised above the heads of the pins or retaining screws, and of a thickness equaling a plate 16 or 18 by the guage, may be polished and burnished. Then grind and adjust the crown, file down and polish the protruding marginal gold to a line with the root and crown. A rubber dam should be used, as it will be almost impossible to make the proper filling otherwise. If the spring of the cleft end be not sufficient to hold the tooth firmly, a fragment of wood in the wire large enough to make the crown go on by smart pressure will answer. *Os Artificiel* or Guillois cement may be placed in the crown, and held in place until it sets.

If the root has been previously pivoted, and therefore more or less funnel-shaped, the filling will be successfully retained by the threads on the pivot and the irregularity of the prepared walls. If this method is carefully and rationally executed by one who can make a good filling with heavy gold, we cannot see in what material sense it will differ from any tooth which has had its nerve destroyed and afterwards filled with gold. In all of the other methods decay will go on. Dr. Mack's method comes nearest to success, but fails on account of the destructible nature of the material used in uniting the crown to the root. If such a necessity should arise, it is perfectly practicable to remove the crown and screw out the pin, as it is also to insert it in the first instance with a fine hole the size of the smallest canal plugger drilled through the entire length, for the discharge from the root, to answer the place of the groove formerly cut along the wooden pivots. However, this latter part is unnecessary, as a root should be restored to a healthy condition before it is pivoted.

---

### SELECTED ARTICLES.

